# The long non-coding RNA *MEG3* plays critical roles in the pathogenesis of cholesterol gallstone

**DOI:** 10.7717/peerj.10803

**Published:** 2021-02-23

**Authors:** Changlin Qian, Weiqing Qiu, Jie Zhang, Zhiyong Shen, Hua Liu, Yongjie Zhang

**Affiliations:** 1 The Second Department of Biliary Surgery, Eastern Hepatobiliary Surgery Hospital, The Second Military Medical University, Shanghai, China; 2 Department of General Surgery, South Campus, Ren Ji Hospital, School of Medicine, Shanghai Jiao Tong University, Shanghai, China

**Keywords:** Cholesterol gallstone, Animal modeling, Library construction, Differential expression analysis, Enrichment analysis, Competing endogenous RNA

## Abstract

**Background:**

Cholesterol gallstone (CG) is the most common gallstone disease, which is induced by biliary cholesterol supersaturation. The purpose of this study is to investigate the pathogenesis of CG.

**Methods:**

Sixteen mice were equally and randomly divided into model group and normal control group. The model group was fed with lithogenic diets to induce CG, and then gallbladder bile lipid analysis was performed. After RNA-seq library was constructed, differentially expressed mRNAs (DE-mRNAs) and differentially expressed lncRNAs (DE-lncRNAs) between model group and normal control group were analyzed by DESeq2 package. Using the cluster Profiler package, enrichment analysis for the DE-mRNAs was carried out. Based on Cytoscape software, the protein-protein interaction (PPI) network and competing endogenous RNA (ceRNA) network were built. Using quantitative real-time reverse transcription-PCR (qRT-PCR) analysis, the key RNAs were validated.

**Results:**

The mouse model of CG was suc cessfully established, and then 181 DE-mRNAs and 33 DE-lncRNAs between model and normal groups were obtained. Moreover, KDM4A was selected as a hub node in the PPI network, and lncRNA *MEG3* was considered as a key lncRNA in the regulatory network. Additionally, the *miR-107-5p/miR-149-3p/miR-346-3-MEG3* regulatory pairs and *MEG3-PABPC4/CEP131/NUMB1* co-expression pairs existed in the regulatory network. The qRT-PCR analysis showed that *KDM4A* expression was increased, and the expressions of *MEG3*, *PABPC4*, *CEP131*, and *NUMB1* were downregulated.

**Conclusion:**

These RNAs might be related to the pathogenesis of CG.

## Introduction

Gallstone disease is a kind of biliary tract diseases, in which cholesterol gallstone (CG) is the most frequent type (Pasternak et al., 2017). CG can be induced by dyslipidemia, overweight, insulin resistance, obesity, and the changes in cholesterol homeostasis (Chen, Kong & Wu, 2015). Genetic factors, lifestyle, and diet are considered to be correlated with the occurrence of CG, especially high-sugar, high-fat, low-fiber, and low-vitamin diets can increase the risk of CG (Di Ciaula et al., 2019; Chang et al., 2019). The formation of CG is based on the imbalances between cholesterol, bile acid, lecithin and other components in bile, which leads to biliary cholesterol supersaturation and crystallization (Di Ciaula, Wang & Portincasa, 2018). CG is common biliary tract disease worldwide, and its incidence has risen sharply over the past decades (Portincasa & Wang, 2017). Therefore, the mechanism of CG should be further explored.

Some RNAs have been reported to be involved in the course of CG. For example, lower serum levels of retinol binding protein 4 (*RBP4*) are detected in CG and are related to gallstone formation, and decreased *RBP4* level was independent on renal function in CG patients  (Wang et al., 2010). The lithogenic diet can result in significantly lower cholecystokinin A receptor (*CCKAR*) and caveolin-3 (*CAV3*) in the gallbladder and lower *CAV3* in the liver, indicating that *CAV3* and *CCKAR* may be implicated in CG (Xu et al., 2014). Through mediating fatty acid and cholesterol metabolism, *miR-122* plays important roles in the development and progression of gallstones (Li et al., 2013; Song et al., 2010). Both *miR-210* and its target ATPase phospholipid transporting 11A (*ATP11A*) are dysregulated in human gallstones, and *ATP11A* expression is negatively correlated with *miR-210* expression in patients with the disease (Yang et al., 2015). However, more RNAs correlated with the pathogenesis of CG still need to be investigated.

Previous studies demonstrate that long non-coding RNA (lncRNA) exerts its biological effects in regulating gene expression by acting as a miRNA sponge (Yuan et al., 2016; Zhang & Huang, 2015). In the present study, the mouse model of CG was established and gallbladder bile lipid analysis was carried out. After the RNA-seq library was constructed, the sequencing data were implemented with a series of bioinformatics analyses to explore the key RNAs and regulatory relationships in CG. Moreover, the key RNAs were validated by quantitative real-time reverse transcription-PCR (qRT-PCR) analysis. Our findings might be helpful to further understand the molecular mechanisms of CG.

## Materials and Methods

### Animal modeling and sample collection

Totally, 16 C57 male mice were purchased from Beijing Vital River Laboratory Animal Technology Co., Ltd. (Beijing, China). The mice were fed with chow diets in specific pathogen free (SPF) laboratory animal room for one week. Then, the mice were randomly divided into model group (*n* = 8) and normal control group (*n* = 8). Mice were housed at 22 ± 2 °C and 60 ± 10% relative humidity in a specific pathogen-free environment, with a 12:12 h light: dark cycle. The model group was fed with lithogenic diets (containing 15% fat, 1% cholesterol, and 0.5% sodium cholate) (Jiangsu Xietong Pharmaceutical Bio-engineering Co., Ltd., Jiangsu, China) for 5 weeks. Meanwhile, the normal control group was fed with chow diets (Wang et al., 2018; Tanaka et al., 2018). During the 5 weeks, food and water were ad libitum. After an overnight fasting, but free access to water, mice were anesthetized with 4% chloral hydrate by intraperitoneal injection. The liver, gallbladder and bile were subsequently isolated, photographed, and kept at −80 °C. The experiments were conducted in accordance with the National Institutes of Health guide for the care and use of laboratory animal, and also approved by the Animal Care and Use Committee in The Second Department of Biliary Surgery, Eastern Hepatobiliary Surgery Hospital.

### Gallbladder bile lipid analysis

According to the manufacturer’s instructions, the changes of total cholesterol (TC), total bile acid (TBA), total bilirubin (TBL), and direct bilirubin (DBL) in bile were detected by corresponding kits (Nanjing Jiancheng Bioengineering Institute, Nanjing, China). Besides, the ratios of TC, phospholipids (PL), and TBA in the model and normal groups were calculated according to the previous reported methods (Carey, 1978). The critical Carey tables were used to calculate the cholesterol saturation index (CSI) to evaluate the cholelithiasis (Carey, 1978).

### RNA-seq library construction and data preprocessing

Using Trizol reagent (Invitrogen, Shanghai, china), total RNA was extracted from four liver tissues from the model group and three liver tissues from the normal control group following the manufacturer’s manual. Then, the integrity and purity of the total RNA were detected by Agarose Gel Electrophoresis and spectrophotometer (Merinton, Beijing, China), respectively. After the RNA-seq library was established using the NEBNext® Ultra™ RNA Library Prep Kit for Illumina® (New England Biolabs, Beverly, MA, USA), library purification, library detection, library quantitation, and cBOT automatic clusters successively were conducted. Furthermore, sequencing was performed using the TruSeq SBS kit v4-HS (Illumina, San Diego, CA, USA).

Quality assessment of the sequencing data was performed by FastQC software (Brown, Pirrung & McCue, 2017) (version 0.10.1, https://github.com/pnnl/fqc). Using Cutadapt software (Chen et al., 2014) (version 1.9.1, https://pypi.org/project/cutadapt/), the adapter sequences, the bases with mass value less than 20 or containing N at the 5′ or 3′ ends, and the reads with length less than 75 bp were filtered out. Subsequently, the clean data was compared with the reference genome using Hisat2 software (Keel & Snelling, 2018) (version 2.0.1, http://daehwankimlab.github.io/hisat2/, default parameters).

### Differential expression analysis and enrichment analysis

Using DESeq2 package (Love, Huber & Anders, 2014) (http://www.bioconductor.org/packages/release/bioc/html/DESeq.html) in R, differential analysis between model group and normal control group was carried out. The mRNAs with the adjusted *p*-value <0.05 and —log_2_ fold change (FC)— >1 were selected as the differentially expressed mRNAs (DE-mRNAs). The lncRNAs with *p*-value <0.05 and —log_2_ FC—>1 were taken as the differentially expressed lncRNAs (DE-lncRNAs). Using pheatmap package (Zhang et al., 2016) (https://cran.r-project.org/web/packages/pheatmap/) in R, hierarchical clustering analysis was performed and clustering heatmap was drew.

Based on clusterProfiler package (Yu et al., 2012) (http://bioconductor.org/packages/release/bioc/html/clusterProfiler.html) in R, Gene Ontology (GO) and Kyoto Encyclopedia of Genes and Genomes (KEGG) enrichment analyses for the DE-mRNAs were implemented. The threshold for selecting the significant results was the *p*-value <0.05.

### Protein–protein interaction (PPI) network analysis

Under the threshold of PPI score (medium confidence) >0.4, PPI network analysis for the DE-mRNAs was conducted using STRING database (Szklarczyk et al., 2017) (http://string-db.org). Combined with Cytoscape software (Kohl, Wiese & Warscheid, 2011) (http://www.cytoscape.org/), the PPI network was constructed. The CytoNCA plug-in (Tang et al., 2015) (parameter: without weight; http://apps.cytoscape.org/apps/cytonca) in Cytoscape software was used to analysis the topology properties of network nodes. The hub nodes (Hsing, Byler & Cherkasov, 2008) were selected according to Degree Centrality (DC), Betweenness centrality (BC), and Closeness centrality (CC) of network nodes.

### Co-expression analysis and prediction of the genes targeted by miRNAs

Pearson correlation coefficients (Schober, Boer & Schwarte, 2018) of the DE-lncRNAs and the DE-mRNAs were calculated. The *r* > 0.95 and *p*-value <0.05 were utilized for screening the significant results. Using miRanda database (Joshi et al., 2016) (http://www.microrna.org), the miRNAs targeting the DE-lncRNAs and the DE-mRNAs were predicted. Under the thresholds of score >1200 and energy <-150, the significant miRNA-lncRNA pairs and miRNA-mRNA pairs were selected.

### Competing endogenous RNA (ceRNA) network analysis and selection of key lncRNAs

Combined with the lncRNA-mRNA co-expression pairs, the miRNA-lncRNA regulatory pairs, and the miRNA-mRNA regulatory pairs, the mRNA-miRNA-lncRNA regulatory relationships were obtained. Using Cytoscape software (Kohl, Wiese & Warscheid, 2011), the ceRNA regulatory network was visualized.

According to the degrees of the lncRNAs in ceRNA regulatory network, the top 8 up-regulated lncRNAs and the top 8 down-regulated lncRNAs separately were selected as the key lncRNAs. Combined the lncRNA-mRNA co-expression pairs, the mRNAs co-expressed with the key lncRNAs were considered as the potential target genes of the key lncRNAs. To obtain the underlying functions of the key lncRNAs, enrichment analysis for these potential target genes was conducted using clusterProfiler package (Yu et al., 2012).

### qRT-PCR analysis

After total RNA was extracted and its integrity and purity were detected, reverse transcription-PCR was performed to synthesize the first-strand cDNA. Then, BeyoFast™ SYBR Green qPCR Mix (2X) (Beyotime, Shanghai, China) and was used for qRT-PCR experiments. The amplification system included: 10 uL BeyoFast™ SYBR Green qPCRMix (2X), 1 uL cDNA template, 0.5 uL forward primer (3 uM), 0.5 uL reverse primer (3 uM), and 8ul RNase-free water. The reaction condition was: 95 °C for 2 min; 95 °C for 15 s, 60 °C for 15 s, 40 cycles; 60 to 95 °C melting curve. Using 2^−^^ΔΔ^^ct^ method, the expression of the targeted genes in relative to GADPH was calculated. The primers used in qRT-PCR experiments were listed in Table 1. Each PCR reaction had three repeats.

**Table 1:** The primers used in quantitative real-time reverse transcription-PCR (qRT-PCR) experiments.

Gene	Primer sequences (5′→3′)
*GAPDH*	GAAGGTCGGTGTGAACGGATTTG
	CATGTAGACCATGTAGTTGAGGTCA
*KDM4A*	ACCCCAGTGCTCGGATCAT
	GGAGGAACGACCTTGGCTA
*PABPC4*	TATACGTGGGCGATTTGCACT
	CGTAGGCATAACCCAGAGAGC
*CEP131*	AGGCAGCGAGCCAAGAAAAA
	CCATGACTGGTTGACCTGTGTA
*NUMB1*	CTCAGAGTTGTGGACGAGAAAAC
	GAGTGGTGCCATCACGACATA
*MEG3*	TGAATGTTGACTGCGTGTGT
	CCTCTCATCTGTCTGCCAATC

## Results

### Animal modeling and gallbladder bile lipid analysis

**Figure 1:**
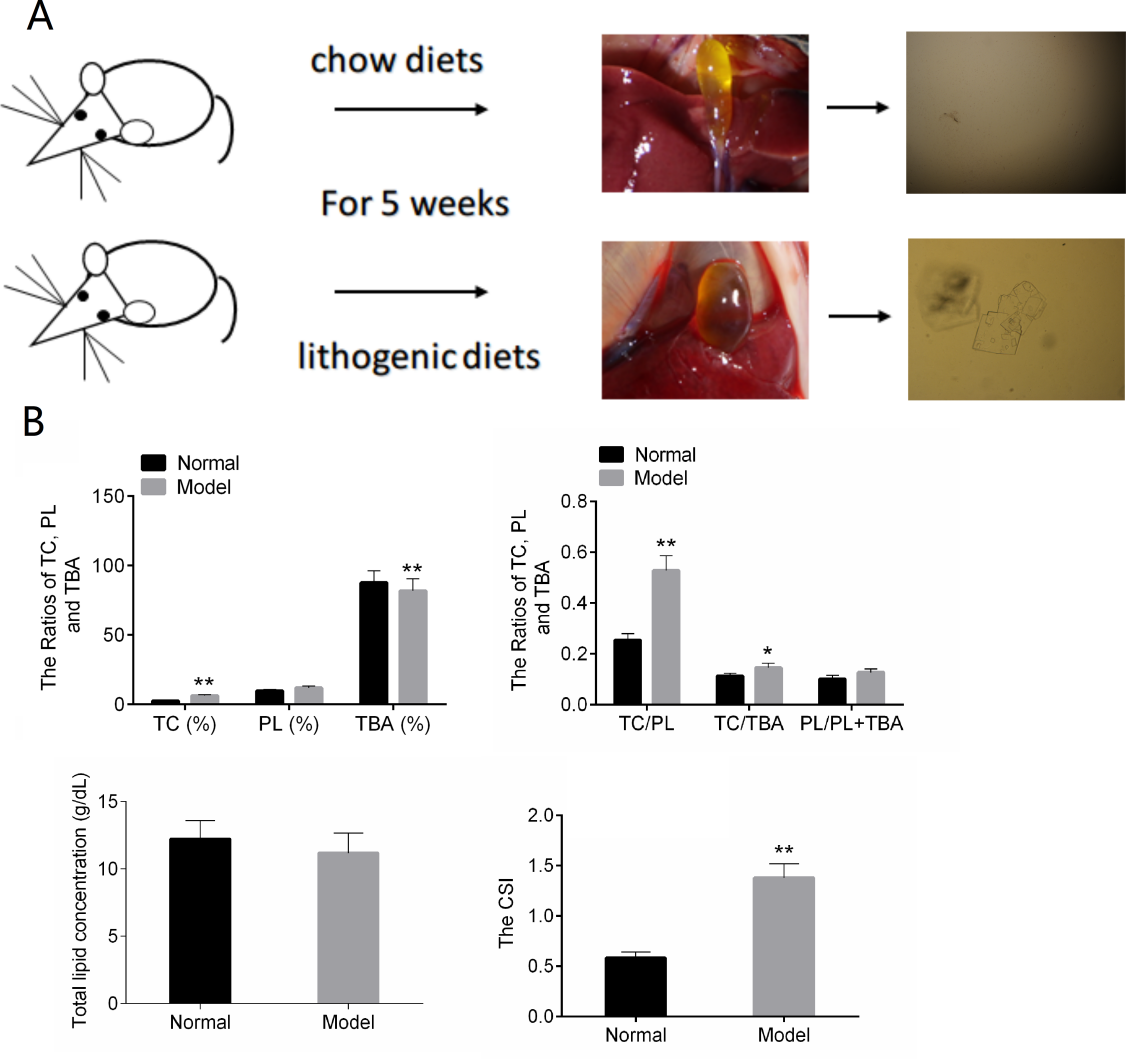
The results of animal modeling and gallbladder bile lipid analysis. (A) The cholesterol crystals in bile of the normal and model groups. (B) The CSI of normal and model groups. TC, total cholesterol; TBA, total bile acid; TBL, total bilirubin; DBL, direct bilirubin; CSI, cholesterol saturation index. * *P* < 0.05; ** *P* < 0.01 vs chow diets group.

In the normal control group, the bile in the gall bladder of the mice was transparent and yellow. After five weeks of lithogenic diets, the gall bladder of the mice in the model group was larger than that in the normal group, and the bile was cloudy and darker (Fig. 1A).The results of Fig. 1B showed that TC, TC/TBA, TC/PL and CSI were increased in the Model group. The TBA was decreased in the Model group (Fig. 1B). These results suggested that the mouse model of CG was successfully established.

### Identification of DE-lncRNAs and DE-mRNAs

**Figure 2:**
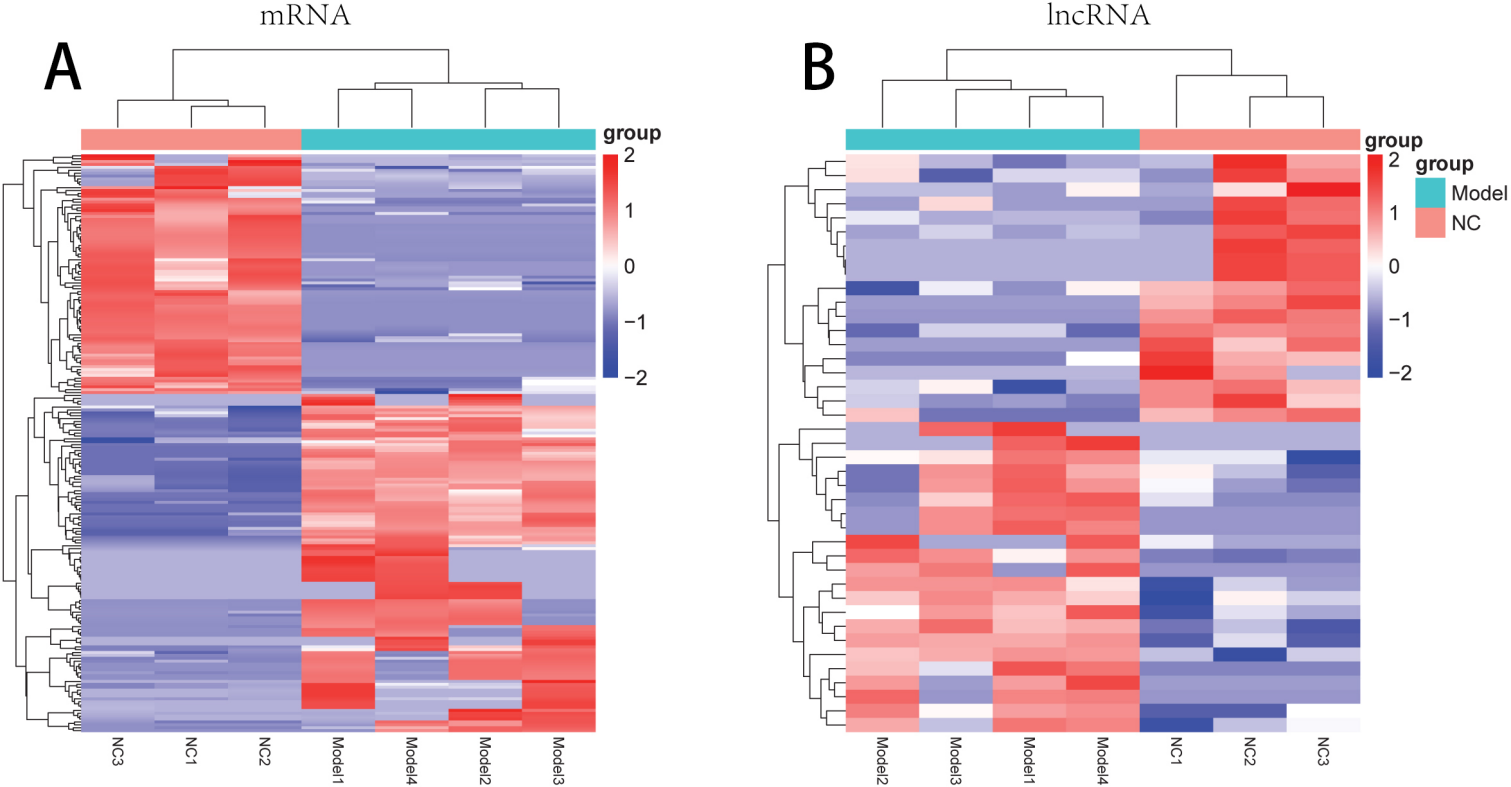
The clustering heatmaps for the differentially expressed mRNAs (A) and the differentially expressed lncRNAs (B). In the sample strips, green and red separately represent model group and normal control (NC) group.

There were 181 DE-mRNAs (including 104 up-regulated mRNAs and 77 down-regulated mRNAs) and 33 DE-lncRNAs (including 17 up-regulated lncRNAs and 16 down-regulated lncRNAs) between model and normal groups. The clustering heatmaps for the DE-lncRNAs and the DE-mRNAs are shown in Fig. 2.

**Figure 3:**
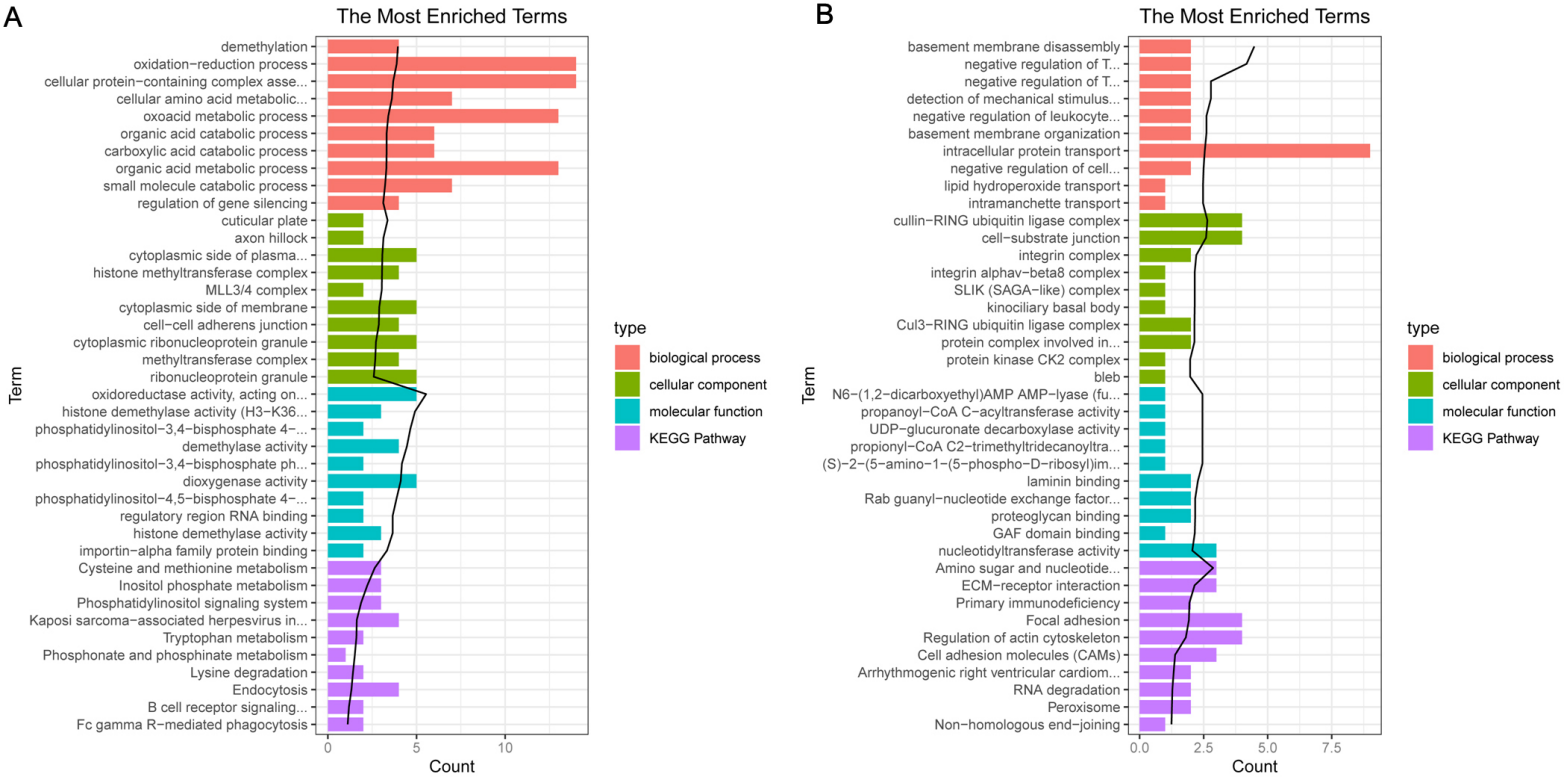
The enrichment results for the differentially expressed mRNAs (top 10 listed). (A) The enrichment results for the up-regulated mRNAs; (B) the enrichment results for the down-regulated mRNAs. Red, green, blue, and purple represent biological process terms, cellular component terms, molecular function terms, and Kyoto Encyclopedia of Genes and Genomes (KEGG) pathways, respectively.

For the up-regulated mRNAs, 419 GO_biological process (BP) terms, 86 GO_cellular component (CC) terms, and 134 GO_molecular function (MF) terms, and eight pathways were enriched (Fig. 3A). For the down-regulated mRNAs, 229 GO_BP terms, 44 GO_CC terms, and 54 GO_MF terms, and seven pathways were enriched (Fig. 3B).

### PPI network analysis

**Figure 4:**
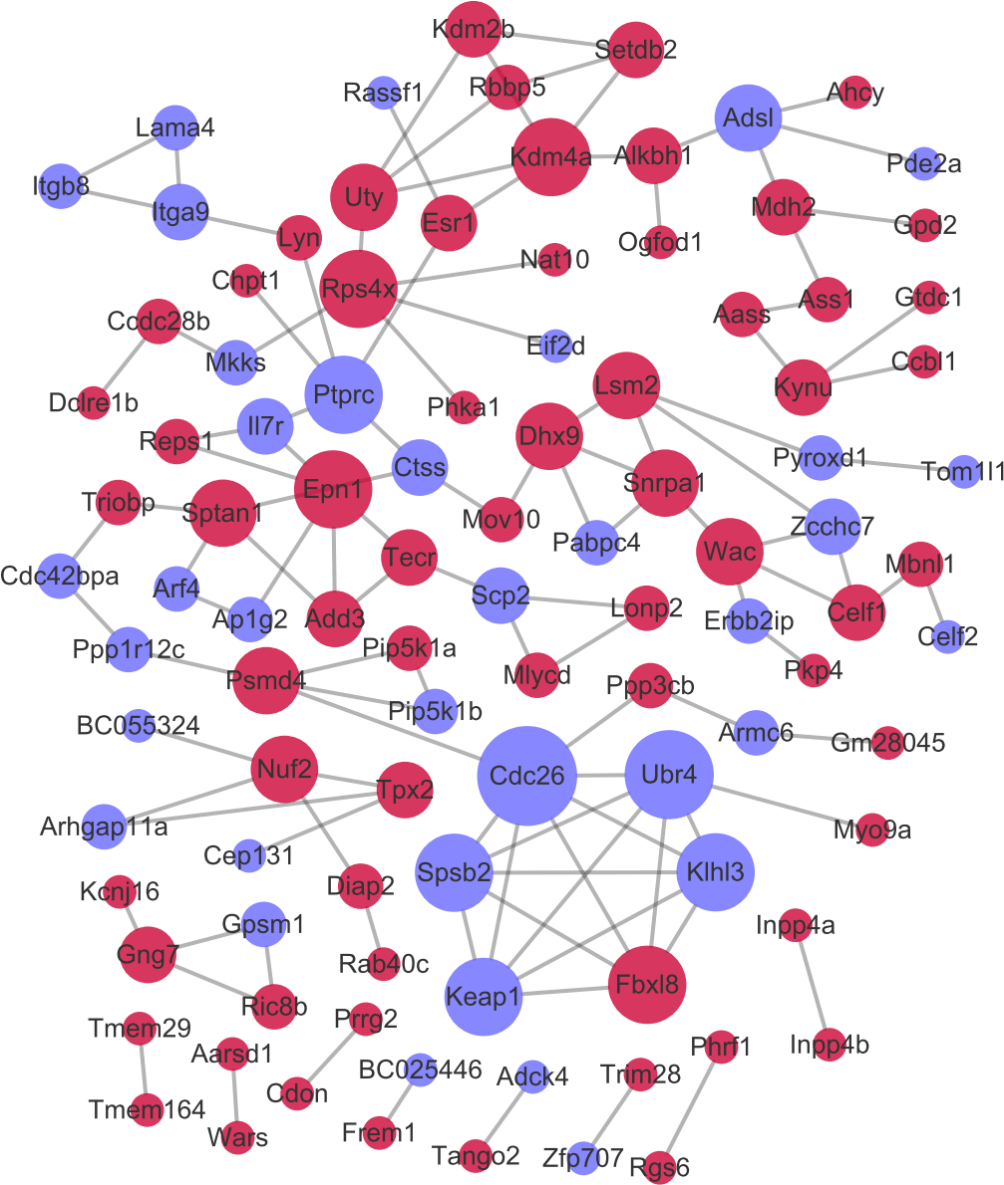
The protein–protein interaction network. Red and green circles separately represent up-regulated mRNAs and down-regulated mRNAs. The node size represents the connectivity degree.

After the PPI pairs for the DE-mRNAs were predicted, PPI network (involving 101 nodes and 116 edges) was constructed (Fig. 4). According to DC, BC, and CC, protein tyrosine 166 phosphatase receptor type C (PTPRC), lysine demethylase 4A (KDM4A), and pectrin alpha, non- 167 erythrocytic 1 (SPTAN1) all were among the top 15 network nodes and were taken as the hub nodes (Table 2).

**Table 2:** The top 15 protein–protein interaction (PPI) network nodes according to degree centrality (DC), betweenness centrality (BC), and closeness centrality (CC).

**Gene_id**	**DC**	**Gene_id**	**BC**	**Gene_id**	**CC**
Cdc26	7	Ptprc	2964	Ptprc	0.032425
Ubr4	6	Ctss	2853	Ctss	0.032415
Kdm4a	5	Kdm4a	2569.333	Esr1	0.032196
Rps4x	5	Esr1	2448	Sptan1	0.032175
Fbxl8	5	Sptan1	2151	Mov10	0.031939
Spsb2	5	Triobp	1740	Kdm4a	0.031928
Ptprc	5	Cdc42bpa	1652	Il7r	0.031857
Klhl3	5	Ppp1r12c	1560	Triobp	0.031746
Keap1	5	Mov10	1560	Lyn	0.031746
Epn1	5	Psmd4	1504	Chpt1	0.031686
Uty	4	Dhx9	1467.667	Epn1	0.031626
Wac	4	Alkbh1	1384	Add3	0.031596
Psmd4	4	Cdc26	1188	Arf4	0.031496
Adsl	4	Adsl	1182	Rassf1	0.031466
Sptan1	4	Uty	988	Dhx9	0.031456

### Co-expression analysis and prediction of the genes targeted by miRNAs

**Figure 5:**
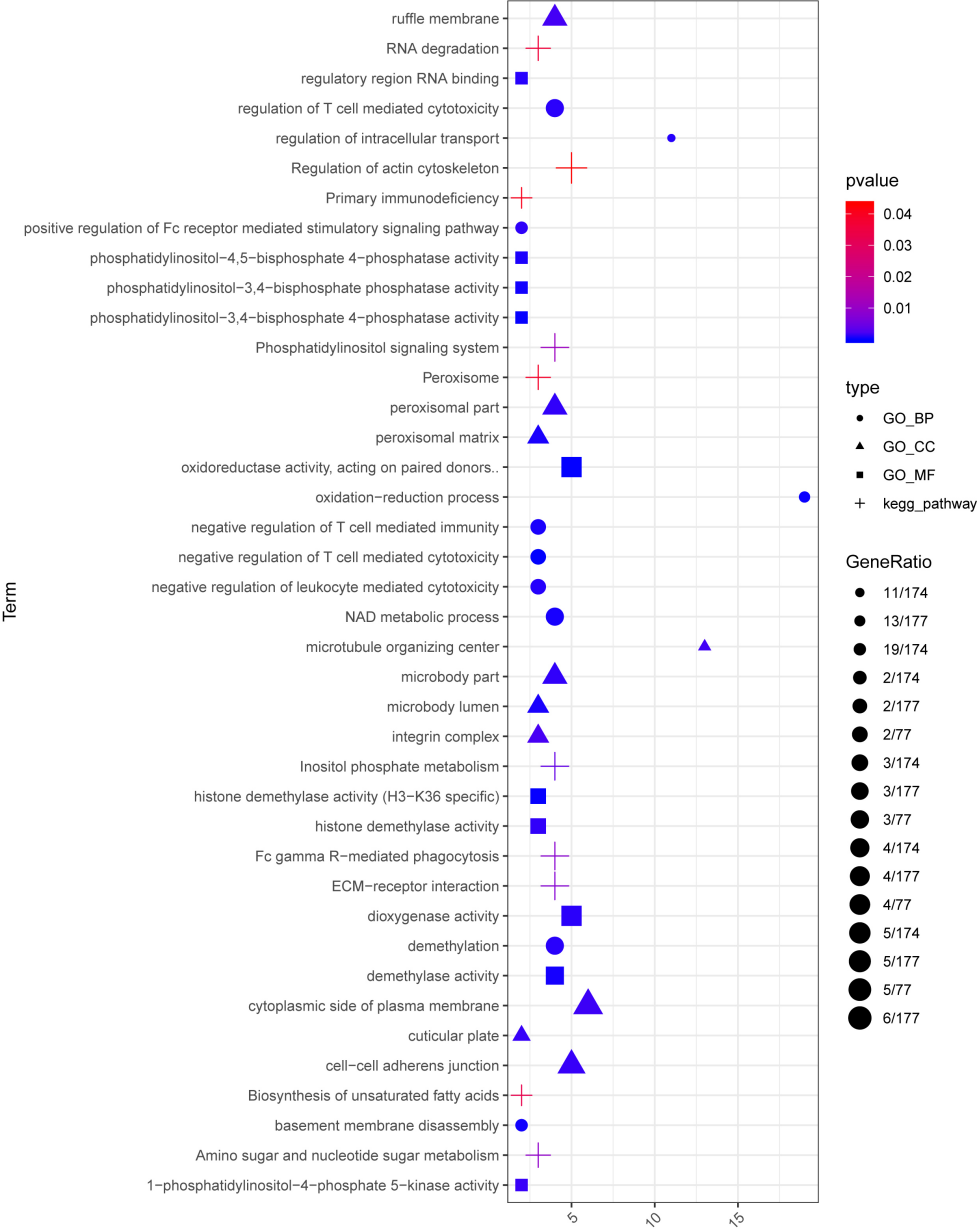
The enrichment results for the lncRNAs implicated in the co-expression pairs. Circles, triangles, squares, and crosses represent Gene Ontology (GO)_biological process (BP) terms, GO_cellular component (CC) terms, GO_molecular function (MF), and Kyoto Encyclopedia of Genes and Genomes (KEGG) pathways, respectively. The color changing from red to blue indicates that the significant *p*-value decreases. The bubble size represents the proportion of the genes involved in one term.

A total of 173 lncRNA-mRNA co-expression pairs were obtained, involving 24 lncRNAs and 96 mRNAs. For each lncRNA implicated in the co-expression pairs, enrichment analysis was conducted for its co-expressed mRNAs. Finally, 457 GO_BP terms, 80 GO_CC terms, and 137 GO_MF terms, and 11 pathways were enriched (Fig. 5). Based on miRanda database, 9320 miRNA-lncRNA pairs (involving 1754 miRNAs and 19 lncRNAs) and 86 miRNA-mRNA pairs (involving 49 miRNAs and 10 mRNAs) were predicted.

### CeRNA network analysis and selection of key lncRNAs

**Figure 6:**
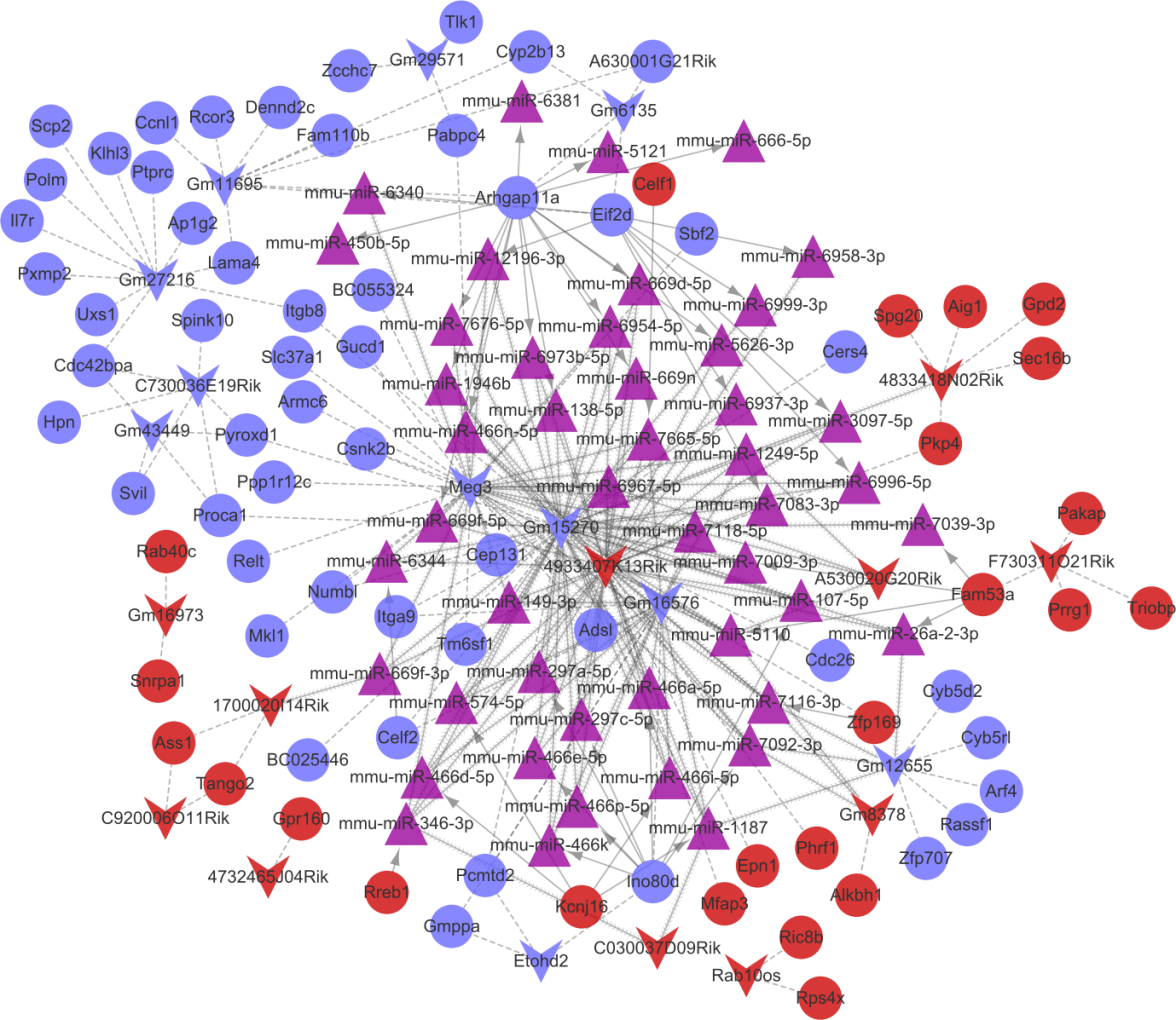
The competingendogenousRNA (ceRNA) network. Red and green separately represent up-regulation and down-regulation. Circles, inverted triangles, and purple regular triangles represent mRNAs, lncRNAs, and miRNAs, respectively. Dotted lines, fish scale lines, and the solid lines with arrows represent the lncRNA-mRNA co-expression pairs, the miRNA-lncRNA regulatory pairs, and the miRNA-mRNA regulatory pairs, respectively. The node size represents the connectivity degree.

Combined with the mRNA-miRNA-lncRNA regulatory relationships, the ceRNA regulatory network (involving 24 up-regulated mRNAs, 53 down-regulated mRNAs, 11 up-regulated lncRNAs, 11 down-regulated lncRNAs, and 47 miRNAs) was built (Fig. 6). There were 42 miRNA-mRNA regulatory pairs, 127 miRNA-lncRNA regulatory pairs, and 115 lncRNA-mRNA co-expression pairs in the ceRNA regulatory network.

**Figure 7:**
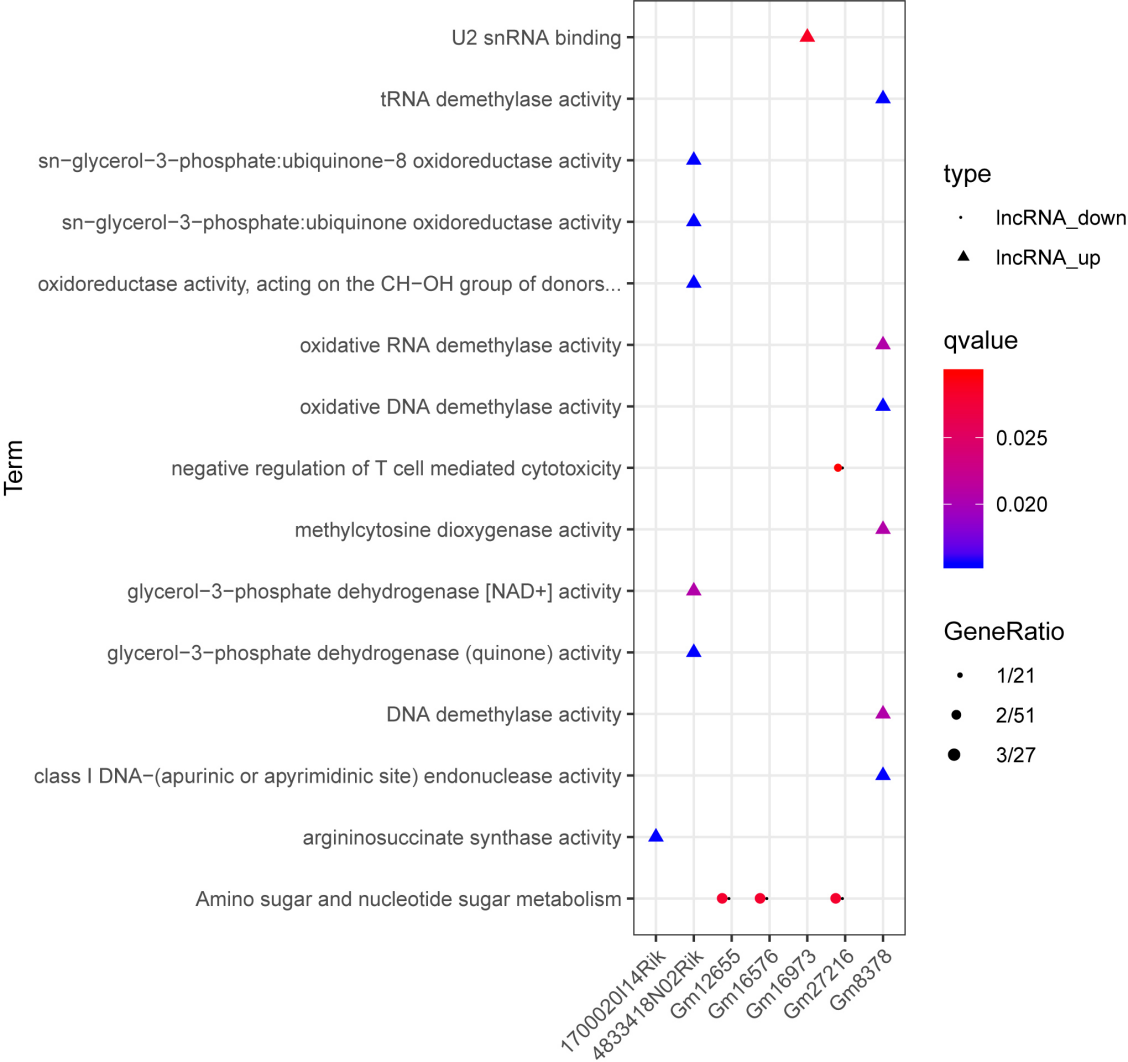
The enrichment results for up-regulated 4833418N02Rik, Gm8378, 1700020I14Rik, and Gm16973, and down-regulated Gm16576, Gm27216, and Gm12655. Triangles and circles represent up-regulated lncRNAs and down-regulated lncRNAs, respectively. The color changing from red to blue indicates that the significant *p*-value decreases. The bubble size represents the proportion of the genes involved in one term.

Based on the degrees of the lncRNAs in the regulatory network, the top eight up-regulated lncRNAs (RIKEN cDNA 4933407K13 gene, *4933407K13Rik*; RIKEN cDNA 4833418N02 gene, *4833418N02Rik*; predicted gene 8378, *Gm8378*; RIKEN cDNA F730311O21 gene, *F730311O21Rik*; RIKEN cDNA A530020G20 gene, *A530020G20Rik*; Opa interacting protein 5, opposite strand 1, *1700020I14Rik*; RAB10, member RAS oncogene family, opposite strand, *Rab10os*; and predicted gene, 16,973, *Gm16973*) and the top eight down-regulated lncRNAs (predicted gene 15,270, *Gm15270*; maternally expressed 3, *MEG3*; RIKEN cDNA C730036E19 gene, *C730036E19Rik*; predicted gene 16,576, *Gm16576*; predicted gene 27,216, *Gm27216*; predicted gene 12,655, *Gm12655*; predicted gene 11,695, *Gm11695*; and predicted gene 6135, *Gm6135*) were screened out as the key lncRNAs. In the regulatory network, the *miR-107-5p/miR-149-3p/miR-346-3p—MEG3* regulatory pairs and *MEG3—PABPC4/CEP131/NUMB1* co-expression pairs existed. To predict the potential functions of these key lncRNAs, enrichment analysis for their co-expressed mRNAs was performed. Moreover, the enrichment results for four up-regulated lncRNAs (*4833418N02Rik*, *Gm8378*, *1700020I14Rik*, and *Gm16973*) and three down-regulated lncRNAs (*Gm16576*, *Gm27216*, and *Gm12655*) are presented in Fig. 7.

### qRT-PCR analysis

**Figure 8:**
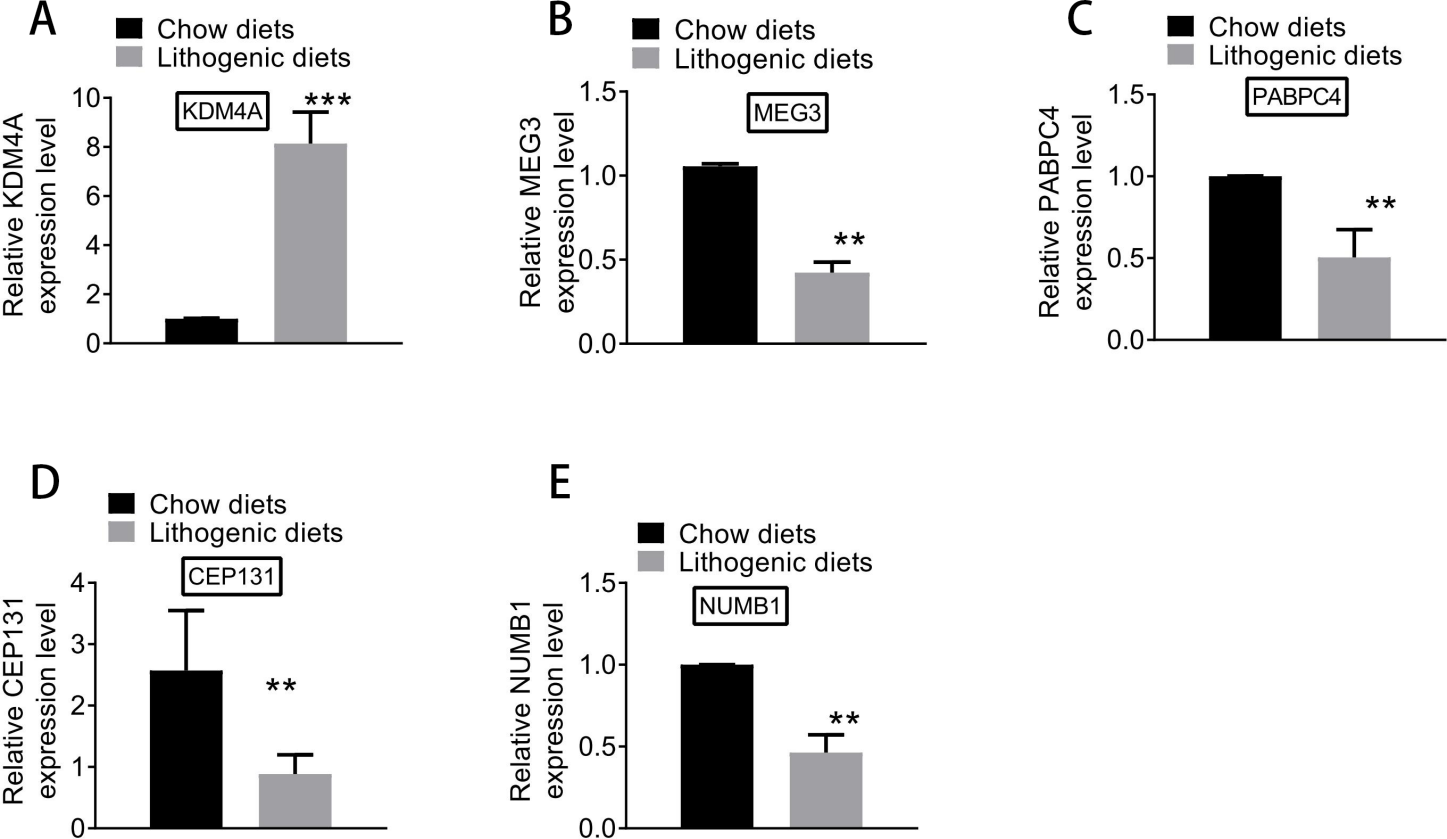
The expression of (A) *KDM4A*, (B) *MEG3*, (C) *PABPC4*, (D) *CEP131*, and (E) *NUMB1* in the model and normal groups.

Based on qRT-PCR experiments, the expression levels of key genes differentially expressed between model and normal groups were examined. As shown in Fig. 8, the expression of *KDM4A* was increased, and the expressions of *MEG3*, *PABPC4*, *CEP131*, and *NUMB1* were decreased in the model group compared with the normal group. The results of qRT-PCR analysis further supported the results of differential expression analysis.

## Discussion

Screening biomarkers in CG is beneficial for CG prevention and treatment, and a number of studies already reported several biomarkers that affect the development of CG. Joshi et al. investigated 4 novel susceptibility loci (*SULT2A1*, *TM4SF4*, *GCKR*, and *CYP7A1*) and confirmed one known locus (*ABCG8*) of CG (Joshi et al., 2016). Th’ng et al. (2018) reported that plasma miR-122, ull-length keratin-18 (flk-18) and caspase-cleaved keratin-18 (cck-18) concentrations were increased in patients with gallstones compared with those without. In the present study, after the mouse model of CG was successfully constructed, 181 DE-mRNAs (including 104 up-regulated mRNAs and 77 down-regulated mRNAs) and 33 DE-lncRNAs (including 17 up-regulated lncRNAs and 16 down-regulated lncRNAs) between model and normal groups were screened. The qRT-PCR experiments confirmed the increased expression of *KDM4A*, as well as the decreased expressions of *MEG3*, *PABPC4*, *CEP131*, and *NUMB1*. In the PPI network, KDM4A was selected as a hub node according to DC, BC, and CC. *KDM4A* expression is reduced during the activation of hepatic stellate cells and its knockdown induces the low expression of *miR-29*, which may provide potential therapeutic approaches for liver fibrosis (Kong et al., 2019). Through recruiting *KDM4*, SBP (S-ribonuclease binding protein) family protein (*BRG1*) activates *β*-catenin target genes and may contribute to hepatic homeostasis and liver repair (Li et al., 2019). These suggested that *KDM4A* might be correlated with the mechanisms of CG.

After the regulatory network was built, the top eight up-regulated lncRNAs and the top eight down-regulated lncRNAs (including *MEG3*) were screened out as the key lncRNAs based on their degrees. *MEG3* suppresses cell proliferation and promotes cell apoptosis in gallbladder cancer, and up-regulating *MEG3* may be applied for inhibiting the deterioration of the tumor (Liu et al., 2016). *MEG3* overexpression in mouse liver can destabilize Shp mRNA and induce cholestatic liver injury via interacting with polypyrimidine tract-binding protein 1 (*PTBP1*) (Zhang et al., 2017). Therefore, *MEG3* might also play roles in the development of CG.

Moreover, the *miR-107-5p/miR-149-3p/miR-346-3p—MEG3* regulatory pairs and *MEG3—PABPC4/CEP131/NUMB1* co-expression pairs were found in the regulatory network. *MiR-107* facilitates hepatic lipid accumulation, causes hyperglycemia and damages glucose tolerance, and thus* miR-107* plays important roles in hepatic lipid accumulation (Bhatia, Pattnaik & Datta, 2016; Joven et al., 2012). The *miR-149* is up-regulated in the HepG2 cells receiving the treatment of long-chain fatty acid (FFA) and contributes to lipogenesis in the HepG2 cells untreated with FFA, therefore, *miR-149* serves as a promising target for treating non-alcoholic fatty liver disease (Xiao et al., 2016; An, Yang & An, 2017). The *miR-346* expression in the peripheral blood mononuclear cells of primary biliary cirrhosis patients is down-regulated in relative to the healthy controls, which may be related to the pathogenesis of the disease (Tan et al., 2014). Zinc finger protein 664 (*ZNF664*) and *PABPC4* variants have different correlations with the high density liptein cholesterol (HDL-C) in adolescents and adults, which may be induced by developmental changes or environmental differences (Middelberg et al., 2012). The rs4660293 in *PABPC4* is related to serum TC, HDL-C, low-density lipoprotein cholesterol (LDL-C) and apolipoprotein A-I (*ApoAI*) levels in the Mulao and Han populations, and a gender-specific correlation is found in these populations (Wu et al., 2015). *CEP131* overexpression promotes cell proliferation and migration in hepatocellular carcinoma through activating the phosphatidylinositol-3 kinase (PI3K)/AKT signaling pathway, therefore, *CEP131* is an oncogene and a candidate prognostic marker in the disease (Liu et al., 2017). Numb in bile in liver mediates cholesterol reabsorption, and the G595D substitution of Numb damages NPC1 like intracellular cholesterol transporter 1 (*NPC1L1*)-associated cholesterol reabsorption in humans with low blood LDL-C (Wei et al., 2014). These indicated that the *miR-107-5p/miR-149-3p/miR-346-3p—MEG3* and *MEG3—PABPC4/CEP131/NUMB1* regulatory axises might be involved in the pathogenesis of CG.

In conclusion, 181 DE-mRNAs and 33 DE-lncRNAs between model and normal groups were identified. Besides, *KDM4A* was implicated in the mechanisms of CG. Furthermore, the *miR-107-5p/miR-149-3p/miR-346-3p—MEG3* and *MEG3—PABPC4/CEP131/NUMB1* regulatory axises might play roles in the development and progression of CG. There are some limitations in the present study. First, because the experimental budget is limited, only 3/16 control animals and 4/16 model animals used for RNA-Seq. Second, the immunohistochemical analysis in liver section of normal and control mice after week 1, 2, 3, 4, 5 was not performed. These will be the part of our future research work. Nevertheless, these results still need to be validated by more rigorous experiments.

##  Supplemental Information

10.7717/peerj.10803/supp-1 Supplemental Information 1 The logFC and p value of DE-mRNA/lncRNA shown in the Tables

10.7717/peerj.10803/supp-2 Supplemental Information 2 Raw numeric data for PCR (Figure 8)

10.7717/peerj.10803/supp-3 Supplemental Information 3 The results of animal modeling, and the cholesterol crystals in bile of the model and normal groups

## Additional Information and Declarations

### Data Availability

The following information was supplied regarding data availability: Raw measurements are available in the Supplemental Files.
