# Supplementary figures and images for: The long non-coding RNA MEG3 plays critical roles in the pathogenesis of cholesterol gallstone
Source: PeerJ. 2021 Feb 23;9:e10803. doi: 10.7717/peerj.10803 (PMC7908887; doi:10.7717/peerj.10803)

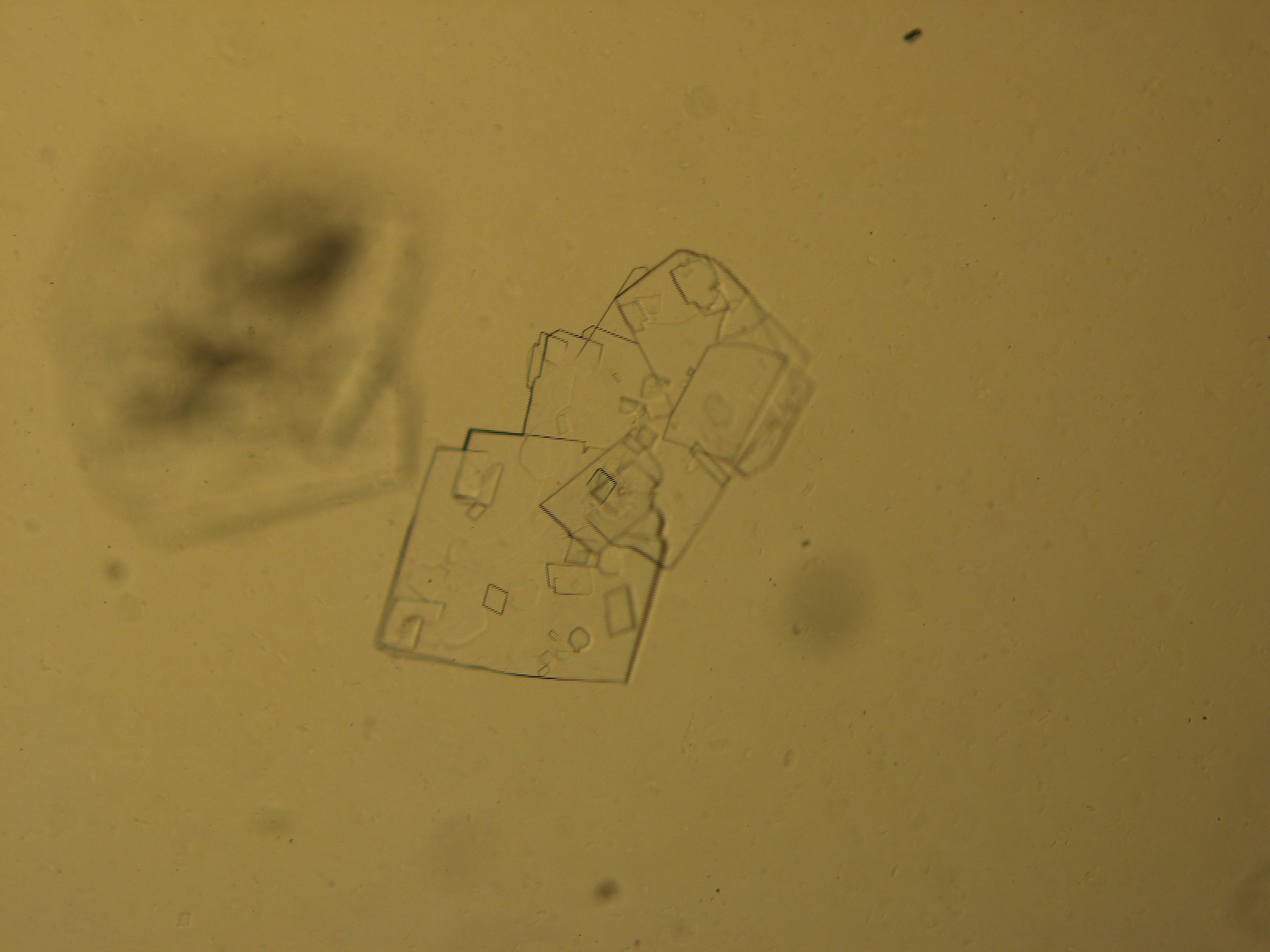

Supplement: Supplemental Information 3 [file peerj-09-10803-s003.zip › supplemental/cholesterol crystals in bile of the model groups.tif]

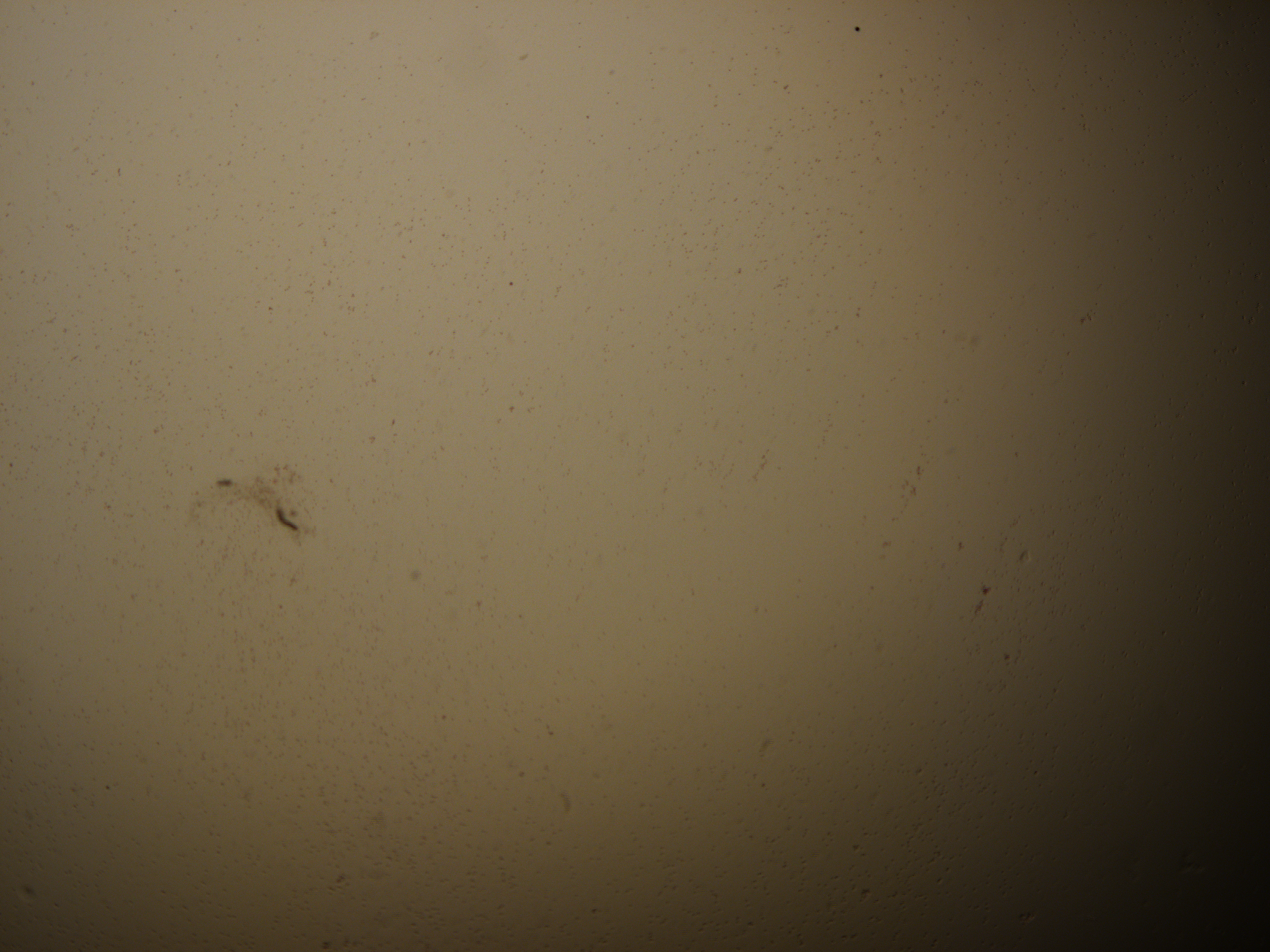

Supplement: Supplemental Information 3 [file peerj-09-10803-s003.zip › supplemental/cholesterol crystals in bile of the normal groups.tif]

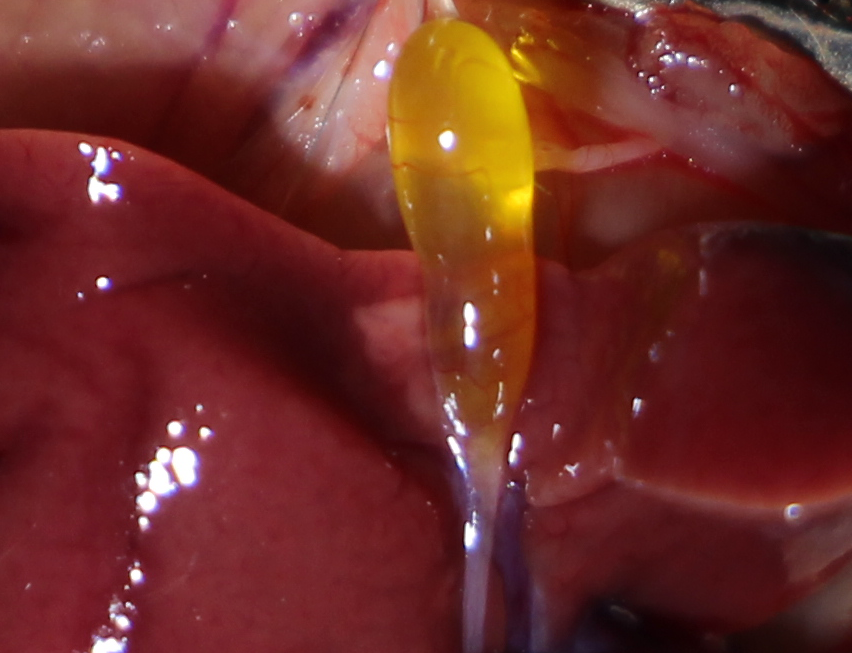

Supplement: Supplemental Information 3 [file peerj-09-10803-s003.zip › supplemental/gallbladder of control group mice.tif]

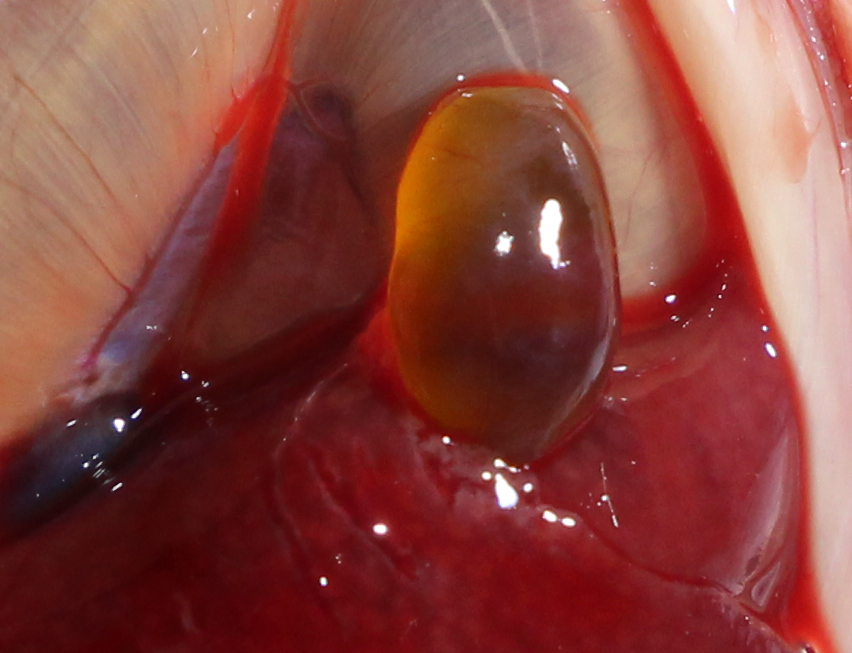

Supplement: Supplemental Information 3 [file peerj-09-10803-s003.zip › supplemental/gallbladder of model group mice.tif]
